# Dipole Relaxation in Semiconducting Zn_2−x_Mg_x_InV_3_O_11_ Materials (Where x = 0.0, 0.4, 1.0, 1.6, and 2.0)

**DOI:** 10.3390/ma13112425

**Published:** 2020-05-26

**Authors:** Tadeusz Groń, Monika Bosacka, Elżbieta Filipek, Sebastian Pawlus, Andrzej Nowok, Bogdan Sawicki, Henryk Duda, Jerzy Goraus

**Affiliations:** 1 August Chełkowski Institute of Physics, University of Silesia in Katowice, 40-007 Katowice, Poland; tadeusz.gron@us.edu.pl (T.G.); sebastian.pawlus@us.edu.pl (S.P.); andrzej.nowok@smcebi.edu.pl (A.N.); bogdan.sawicki@us.edu.pl (B.S.); henryk.duda@us.edu.pl (H.D.); 2 Faculty of Chemical Technology and Engineering, Department of Inorganic and Analytical Chemistry, West Pomeranian University of Technology in Szczecin, 71-065 Szczecin, Poland; bossm@zut.edu.pl (M.B.); elafil@zut.edu.pl (E.F.)

**Keywords:** electrical properties, dielectric spectroscopy, relaxation processes

## Abstract

This paper reports on the electrical and broadband dielectric spectroscopy studies of Zn_2−x_Mg_x_InV_3_O_11_ materials (where x = 0.0, 0.4, 1.0, 1.6, 2.0) synthesized using a solid-state reaction method. These studies showed *n*-type semiconducting properties with activation energies of 0.147–0.52 eV in the temperature range of 250–400 K, symmetric and linear I–V characteristics, both at 300 and 400 K, with a stronger carrier emission for the matrix and much less for the remaining samples, as well as the dipole relaxation, which was the slowest for the sample with x = 0.0 (matrix) and was faster for Mg-doped samples with x > 0.0. The faster the dipole relaxation, the greater the accumulation of electric charge. These effects were analyzed within a framework of the DC conductivity and the *Cole–Cole fit* function, including the solid-state density and porosity of the sample. The resistivity vs. temperature dependence was well fitted using the parallel resistor model. Our ab initio calculations also show that the bandgap increased with the Mg content.

## 1. Introduction

It is known from the literature that double vanadates of the general formula M^II^_2_M^III^V_3_O_11_, i.e., M_2_FeV_3_O_11_ (M = Mg, Zn, Ni), M_2_CrV_3_O_11_ (M = Mg, Zn), and M_2_InV_3_O_11_ (M = Mg, Zn, Co), crystallize in the triclinic system [1,2,3,4,5,6]. These compounds may be used as catalysts or photo-catalysts in various chemical processes. In particular, complex metal oxides based on iron ions are the most attractive objects for investigation. For example, in functional hexaferrite BaFe_12−x_DI_x_O_19_ (DI = Al^3+^, In^3+^ 0.1 ≤ x ≤ 1.2) solid solutions with an increase in Al^3+^ and In^3+^ ion concentrations, the natural ferromagnetic resonance frequency is shifted from 51 GHz to 61 GHz and from 50.5 GHz to 27 GHz, respectively [7]. In M-type hexaferrite BaFe_12−x_In_x_O_19_ (x = 0.1, 1.2) samples, an influence of structural parameters on the temperature behavior of Fe^3+^(i)–O^2−^–Fe^3+^(j) (i, j = 1, 2, 3, 4, 5) indirect superexchange interactions was established [8]. In turn, in nanosized ferrites of Sr_0.3_Ba_0.4_Pb_0.3_Fe_12_O_19_/(CuFe_2_O_4_)_x_ (x = 2, 3, 4, and 5), non-linear behavior of the microwave characteristics was observed [9].

A solid solution of a Zn_2−x_Mg_x_InV_3_O_11_ composition was found to form in the Zn_2_InV_3_O_11_–Mg_2_InV_3_O_11_ system [10]. The phases with x = 0.0, 0.4, 1.0, 1.6, and 2.0 were obtained using solid-state reactions. The solid solution under study crystallized in the triclinic system. Its unit cell parameters *a* and *b* increased, while the unit cell parameter *c* decreased with an increasing magnesium content [10]. The solid-state density values obtained experimentally (*d*_exp_) and calculated (*d*_cal_) based on X-ray diffraction (XRD) data decreased as a function of the degree of Mg^2+^ ions’ incorporation in the crystal lattice of Zn_2_InV_3_O_11_ [10], where a good agreement was shown between *d*_exp_ and *d*_cal_. The level of porosity of *p*_l_ ≈ 1.67% determined using the formula *p*_l_ = (1 − *d*_exp_/*d*_cal_) did not depend on the magnesium content x. Similar behavior was found for M_2_FeV_3_O_11_ compounds (where M = Mg, Ni, Pb) [11]. Scanning electron microscopy (SEM) images of Zn_2−x_Mg_x_InV_3_O_11_ samples with x = 0.0, 1.0, and 2.0 showed almost identical shapes and sizes to the crystallites of the compounds (x = 0.0 and 2.0), while the crystallites of the solid solution (x = 1.0) had a similar shape but were much larger [10]. The infrared spectroscopy measurements showed that all recorded spectra were similar and contained the stretching vibrations of V–O bonds in highly distorted VO_4_ and VO_5_ polyhedra in the wavenumber range of 1000–900 cm^−1^ [1,2,5,12,13]. Next, in the absorption band covering the range 900–400 cm^−^^1^, the stretching vibrations were ascribed to the moderately distorted VO_4_ tetrahedra or to stretching vibrations in ZnO_x_ and MgO_x_ [1,13,14,15,16]. Recently, the preliminary results of magnetic and dielectric measurements of Zn_2−x_Mg_x_InV_3_O_11_ phases with x = 0.0, 0.4, and 1.6 showed diamagnetic behavior above room temperature and a strong increase in the relative electrical permittivity as the magnesium content increased [17].

Here, we present the results of electrical measurements and broadband dielectric spectroscopy studies of Zn_2−x_Mg_x_InV_3_O_11_ materials (where x = 0.0, 0.4, 1.0, 1.6, 2.0). The samples under study were almost identical to those described in Bosacka and Filipek [10], which contains all the details of the conducted synthesis, morphology, and X-ray phase analysis. The main motivation of this work was to examine the dielectric relaxation in the frequency representation using the sum of the conductivity part and the *Cole–Cole fit* function.

## 2. Experimental and Calculation Details

### 2.1. Synthesis and Phase Analysis

Preliminary studies of the reactions occurring in the ZnO–MgO–V_2_O_5_–In_2_O_3_ system allowed for synthesizing a series of solid solutions with the formula Zn_2__−x_Mg_x_InV_3_O_11_ (x = 0.0, 0.4, 1.0, 1.6, 2.0) [10]. Synthesis of the solid solution was performed using the solid-state reaction method described in Bosacka and Filipek [10]. XRD analysis of all samples showed them to be monophasic. XRD patterns of single-phase samples containing Zn_2__−x_Mg_x_InV_3_O_11_ were the same as those recorded in Bosacka and Filipek [10]. Tests of all samples synthesized in this study also confirmed that a continuous solid solution with the general formula Zn_2__−x_Mg_x_InV_3_O_11_ crystallized in the triclinic system and the parameters of its unit cell *a* and *b* increased, while the parameter *c* decreased with increasing magnesium content.

**Figure 1:**
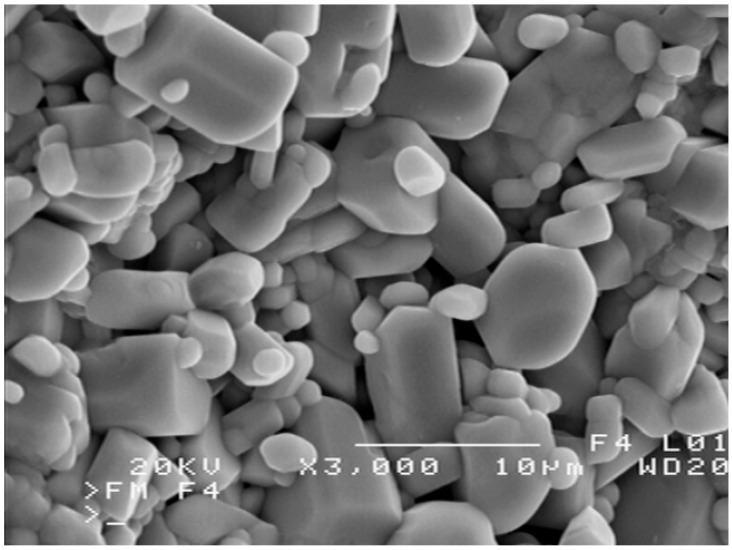
SEM image of ZnMgInV_3_O_11._

The sample morphology observed on the scanning electron microscopy images showed that the crystal phases were irregular polyhedra and their average size was about 1–5 μm. As an example, Figure 1 shows an SEM image of a sample containing Zn_2__−x_Mg_x_InV_3_O_11_ with x = 1. The results of the experimental determination of the crystals’ composition using Energy Dispersive X-ray (EDX) analysis of this monophase sample showed that the Zn:Mg:In:V ratios were near 1:1:1:3 (on average: 17.3 at% Zn, 15.5 at% Mg, 16.5 at% In, and 50.7 at% V), which corresponded to the values from the formula ZnMgInV_3_O_11_.

**Figure 2:**
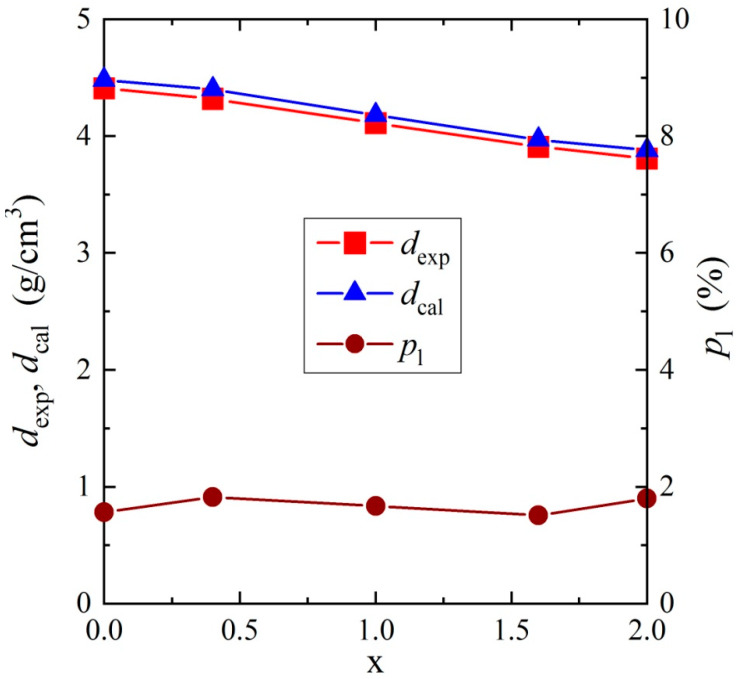
Experimental, *d*_exp_, and calculated, *d*_cal_, solid-state density and porosity level, *p*_l_, as a function of the content x of Zn_2−x_Mg_x_InV_3_O_11_. Calculated data is taken from Bosacka and Filipek [10].

The densities of the samples (*d*_exp_) were determined using a gas pycnometer (Ultrapyc 1200e, Quantachrome Instruments, Boynton Beach, FL, USA) and argon with a 5 N purity was used as the pycnometric gas. Its dependence on x in Figure 2 is the same as in Bosacka and Filipek [10].

### 2.2. Electrical Measurements

The electrical conductivity *σ*(T) in the temperature range of 80–400 K and the I–V characteristics at 300 and 400 K were measured using the DC method using a KEITHLEY 6517B Electrometer/High Resistance Meter (Keithley Instruments, LLC, Solon, OH, USA). The thermoelectric power *S*(T) was measured in the temperature range of 300–400 K using a Seebeck Effect Measurement System (MMR Technologies, Inc., San Jose, CA, USA). The broadband dielectric spectroscopy measurements were carried out using pellets that were polished and sputtered with (≈80 nm) Ag electrodes in a frequency range from 2 × 10^2^–2 × 10^6^ Hz using an LCR HITESTER (HIOKI 3532-50, New York, NY, USA) and in the temperature range 80–400 K. For the electrical measurements, the powder samples were compacted in a disc form (10 mm in diameter and 1–2 mm thick) using a pressure of 1.5 GPa; then, they were sintered for 2 h at 923 K. The electrical and thermal contacts were made using a silver lacquer mixture (Degussa Leitsilber 2000, is Degussa Gold und Silber, Munich, Germany).

### 2.3. Density Functional Calculations

We performed the band structure calculations based on the density functional theory (DFT) using CRYSTAL software (CRYSTAL17, University of Turin, Turin, Italy) [18] for three representatives of the Zn_2__−x_Mg_x_InV_3_O_11_ series (x = 0.0, 1.0, 2.0) since due to the method used, we could only substitute particular atoms in the unit cell, and hence fractional substitution was not possible. The CRYSTAL software uses a Gaussian type basis set: for In, we used the basis employed in Rothballer et al. [19], and for the remaining lighter elements, we used the double-zeta basis, as published in Vilela Oliveira et al. [20]. We used the exchange-correlation potential as proposed in Perdew et al. [21] and 260 *k*-points in the irreducible wedge of the Brillouin zone. We relaxed the atomic positions (using default criteria in CRYSTAL) but the lattice parameters were taken from the XRD measurements. The unit cells of the considered compounds were large and low-symmetry with 17 inequivalent Wyckoff positions. Hence, the lattice dynamics or elastic parameters were not studied here; we were only interested in the change of the semiconducting bandgap width relative to the Mg content.

## 3. Results and Discussion

### 3.1. Electrical Properties and Density of States

**Figure 3:**
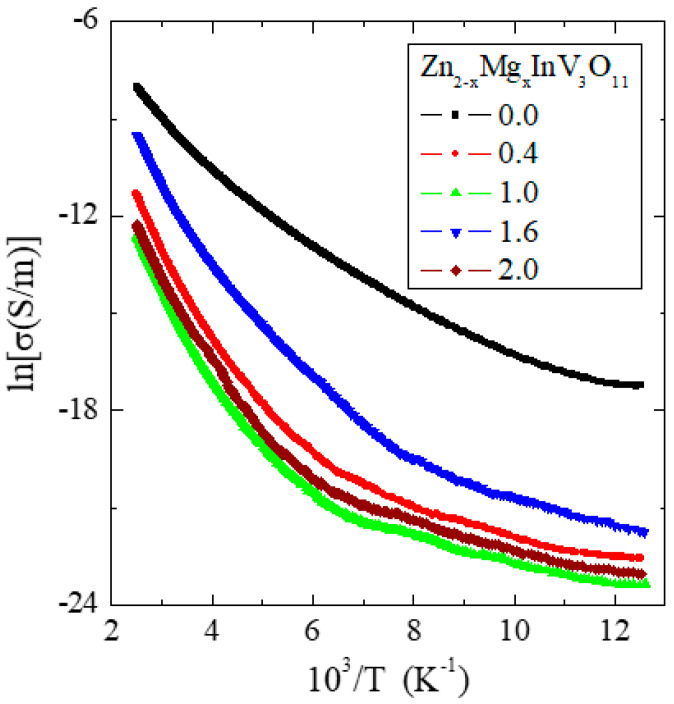
Electrical conductivity (lnσ) vs. reciprocal temperature 10^3^/T of Zn_2−x_Mg_x_InV_3_O_11_.

**Figure 4:**
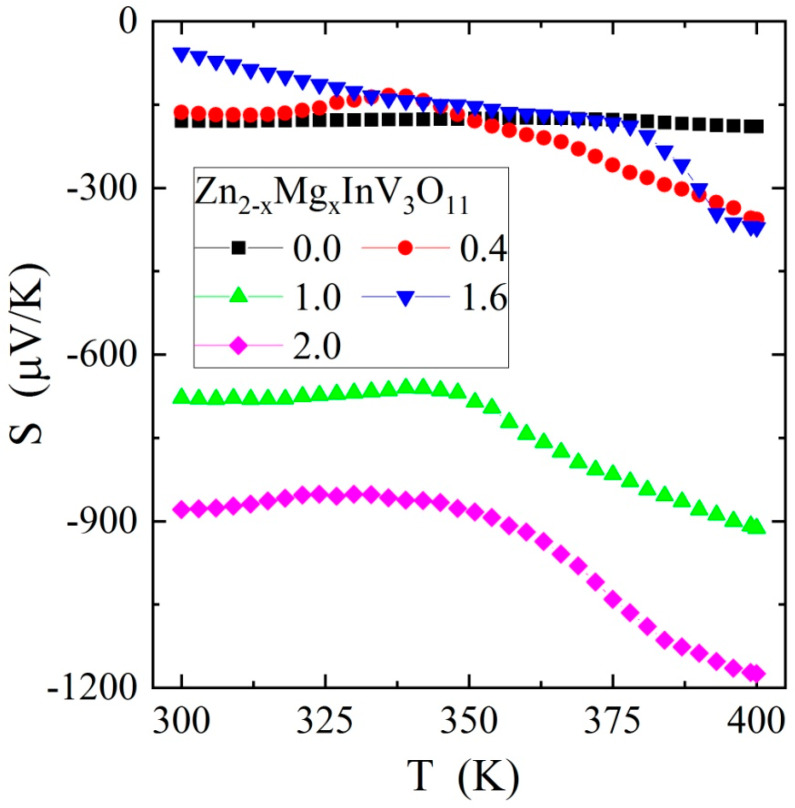
Thermoelectric power S vs. temperature T of Zn_2−x_Mg_x_InV_3_O_11_.

**Figure 5:**
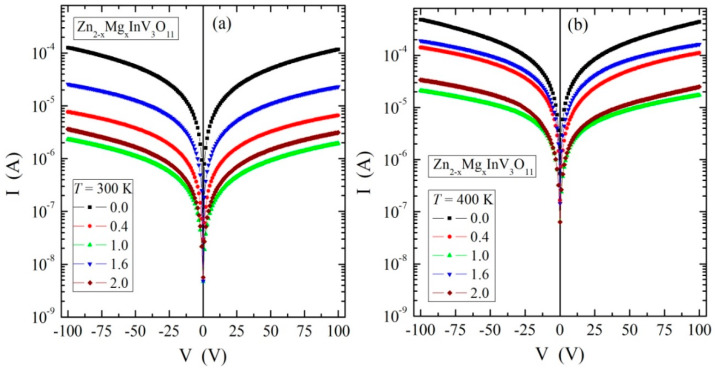
The I–V characteristics at 300 (**a**) and 400 K (**b**) of Zn_2−x_Mg_x_InV_3_O_11_.

The results of the electrical measurements of the solid solution of Zn_2−x_Mg_x_InV_3_O_11_ (x = 0.0, 0.4, 1.0, 1.6, and 2.0) composition showed semiconducting properties with an activation energy of 0.2–0.3 eV in the temperature range of 250–400 K, i.e., in an intrinsic region (Figure 3) and with *n*-type conductivity (Figure 4). The I–V characteristics, measured at 300 and 400 K (Figure 5), showed symmetric and linear behavior with more carrier emissions for the matrix (Zn_2_InV_3_O_11_) and much less for the remaining samples.

In Figure 5, the 300 (a) and 400 K (b) plots of the matrix (x = 0.0) compared to the other phases (x > 0.0) indicated the existence of vanadium ions with mixed valences in the matrix. With increased magnesium content, this mixed valence of vanadium ions was reduced. Generally, small values of the electrical conductivity in the temperature range of 80–400 K and low electron emissions of the phases under study (Figure 3 and Figure 5) indicated a good dielectric behavior, which may have been due to the existence of centers trapping current carriers lying in the deep donors. The energy required for the creation of a vacancy depends on the bond type. The stronger the bond, the higher the energy needed for the creation of a vacancy. Similar behavior in AgY_1−x_(Gd,Nd)_x_(WO_4_)_2_ was observed [22].

It is worth noting that if the activation law properly described the resistivity vs. temperature dependence of these materials, the ln(σ) vs. 1/T curve shown in Figure 3 should be a straight line. We see that this is not the case. A simple explanation can be provided based on the parallel resistor model (PRM) [23,24], where we assume that the resistivity vs. temperature dependence *ρ*(T) of these materials consists of two parts, which are connected in parallel. One is described by the activation law $\rho_{0}\exp\left(\frac{\Delta }{2kT}\right)$ and the second is constant relative to temperature $\rho_{p}=const$. Here, Δ denotes a bandgap in the density of states between the conduction and valence states and *k* is the Boltzmann constant. This model (Equation (1)), with $\rho_{0}$, $\rho_{p}$, and Δ as fitting parameters, was recently successfully employed to explain the semiconducting properties of cisplatin [25].

(1) $$\rho \left(T\right)=\frac{\rho_{p}\rho_{0}\exp\left(\frac{\Delta }{2kT}\right)}{\rho_{p}+\rho_{0}\exp\left(\frac{\Delta }{2kT}\right)}$$

**Figure 6:**
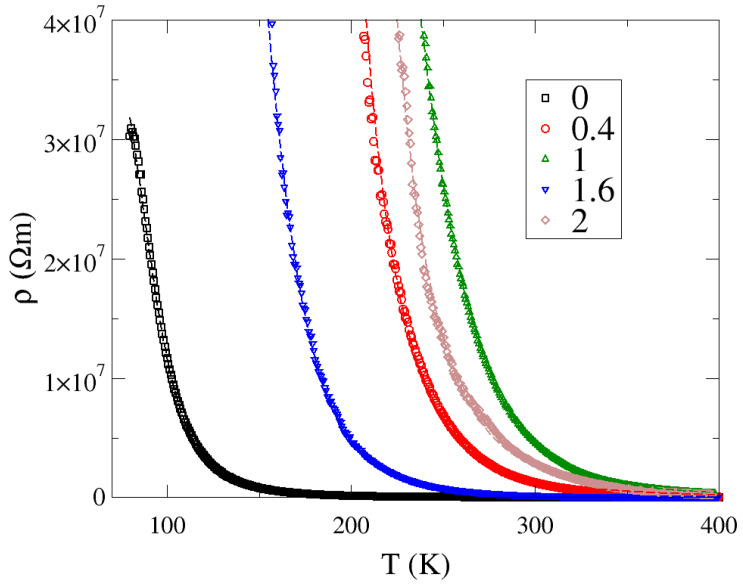
The resistivity of Zn_2−x_Mg_x_InV_3_O_11_ (empty symbols) fitted using the parallel resistor model [23].

In Figure 6, we show the measured resistivity vs. temperature (empty symbols) with the PRM fit (dashed line). We see that this model almost perfectly describes the resistivity vs. temperature curve of Zn_2__−x_Mg_x_InV_3_O_11_ series over a broad temperature range. The fit parameters are given in Table 1. The pristine Zn_2_InV_3_O_11_ compound had the narrowest bandgap of about 0.154 eV, whereas compositions where Mg replaced Zn atoms had wider bandgaps of about 0.3–0.517 eV. Moreover, the width of the bandgap did not change monotonically with Mg content for those compounds. However, it should be noted that the parallel resistivity *ρ*_p_ also changed significantly, over four orders of magnitude. This *ρ*_p_ represents the current leakage, which might be related to grain size, off-stoichiometry, and sample synthesis conditions, and not to the inherent properties of these compounds. Probably, it is also strongly affected by the pressure used to compress the samples during their synthesis.

**Figure 7:**
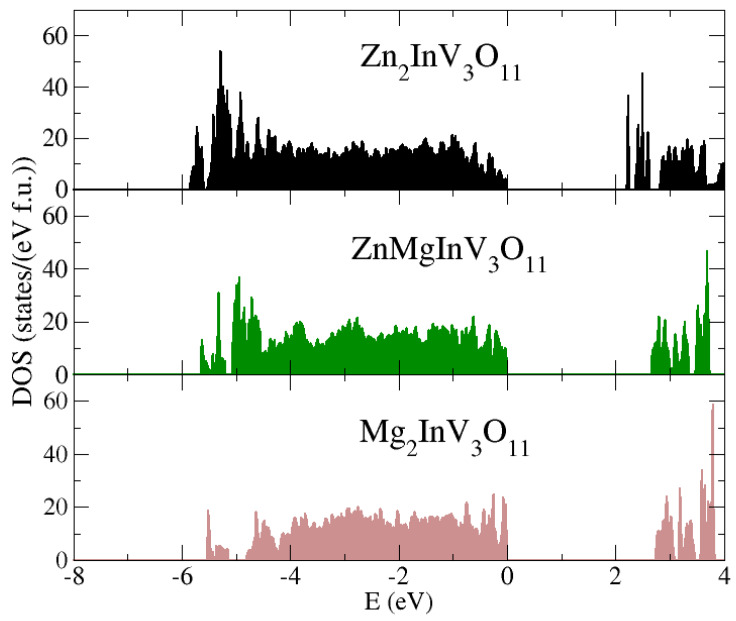
The total density of states (DOS) calculated for Zn_2−x_Mg_x_InV_3_O_11_ (x = 0, 1, 2).

We determined the total density of states based on density functional theory (DFT) calculations (see Figure 7) for three members of the series Zn_2__−x_Mg_x_InV_3_O_11_ (x = 0, 1, 2). The obtained ground state was non-magnetic, in agreement with our preliminary experimental data mentioned in the introduction. We see that width of the bandgap increased with Mg content and the narrowest bandgap was observed for the pristine Zn_2_InV_3_O_11_ compound. This agrees well with our resistivity data. One might notice that the bandgap obtained from the DFT calculations was wider than the one obtained from fitting the experimental *ρ*(T) curve. This is a little bit atypical, as usually the DFT calculations underestimate the width of the bandgap. Here, we explain this phenomenon in terms of the complicated structure of the Zn_2−x_Mg_x_InV_3_O_11_ unit cell, as well as the residual states that are formed in the bandgap due to atomic disorder [26].

### 3.2. Dielectric Results

**Figure 8:**
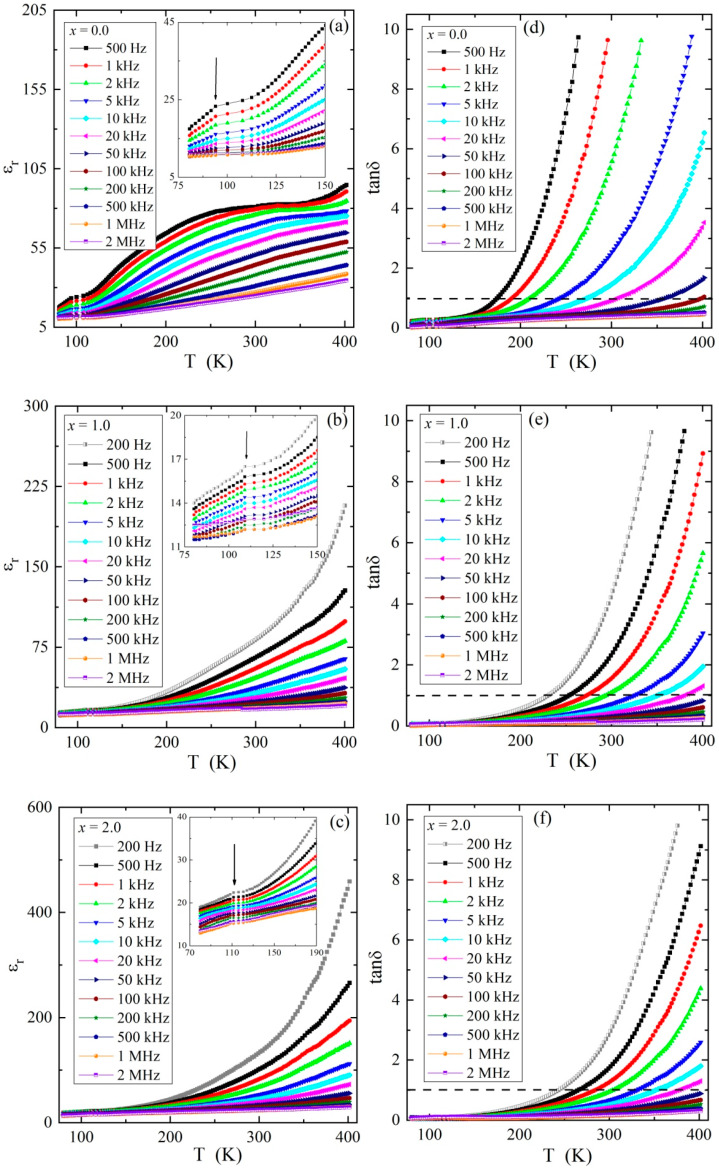
Dielectric constant ε_r_ (x = 0.0 for (**a**), x = 0.1 for (**b**), x = 0.2 for (**c**)) and loss tangent tanδ (*x* = 0.0 for (**d**), x = 0.1 for (**e**), x = 0.2 for (**f**)) vs. temperature T of Zn_2−x_Mg_x_InV_3_O_11_ in the frequency range 200 Hz to 2 MHz. Insets: There was a change in the T-dependence of ε_r_ in the vicinity of 94 K (for x = 0.0 in (**a**)), 109 K (for x = 1.0 in **b**), and 111 K (for x = 2.0 in (**c**)), which are marked with vertical arrows. Horizontal dashed lines in the tanδ vs. T dependences indicate the value of tanδ = 1.

The results of the broadband dielectric spectroscopy measurements of Zn_2−x_Mg_x_InV_3_O_11_ for the representative x = 0.0, 1.0, and 2.0 displayed in Figure 8a–f showed a strong increase in the relative electrical permittivity, ε_r_, above 150 K, irrespective of the magnesium content in the sample. On the other hand, the maximum value of ε_r_ for the lowest frequencies (marked with vertical arrows in Figure 8a–c) increased with the magnesium content. With the increase in the frequency of the electric field, both ε_r_ and the loss tangent tanδ strongly decreased. In particular, a high loss was observed at low frequencies, which is characteristic of Joule–Lenz-type losses (Figure 8d–f). It should be noted that tanδ at 500 Hz crossed the value of 1 at ≈175 K for x = 0, whereas for samples with a higher concentration of magnesium, this temperature increased to ca. ≈250 K for x = 1.0 and x = 2.0 (see horizontal dashed lines in Figure 8d–f).

The above-mentioned dielectric properties could be interpreted as being related to a relaxation process, such as in Maxwell-Wagner [27] or Jonscher [28], which was strongly obscured by the DC conductivity with an activation energy of 0.2–0.3 eV in the intrinsic region. A similar relaxation process has been found in Sr_2_InV_3_O_11_ [29], M_2_FeV_3_O_11_ (M = Mg, Zn, Pb, Co, Ni) [11], and Nb_6_VSb_3_O_25_ compounds with an activation energy of 0.75 eV [30,31]. From Figure 8, one can see that the variation in ε_r_ strongly depended on the content of magnesium ions, which reduced the number of vanadium ions with mixed valence. Finally, this led to the accumulation of electric charge in the deep trapping centers [32] lying under the bottom of the conduction band. A natural source of these traps can be grain boundaries with depletion layers of adjacent grains, as it has been observed for ZnO varistors [33], Nb_2_VSbO_10_ compounds [34], and some copper/cobalt and rare-earth metal tungstates [35], as well as for (Co,Mn)Pr_2_W_2_O_10_ materials [36].

### 3.3. Dielectric Analysis

It must be noted that, as already presented above, both the ε_r_ and tanδ as functions of temperature provide only limited information about the electric properties of the materials. Let us start the dielectric analysis of the temperature dependence of the dielectric permittivity, ε_r_(T), of two representative samples with x = 0.0 and 2.0, for which the transition region is marked with an arrow in Figure 8a,c. As can be seen, ε_r_ declined with a decrease in temperature and a decrease in frequency for both samples with x = 0.0 and 2.0. However, this relationship was nonlinear in nature, which can indicate the existence of dipole relaxation. Unfortunately, this weekly visible dipole relaxation was impossible to analyze in the plots with varying temperatures. Moreover, there was a change in the *T*-dependence of ε_r_ in the vicinity of 100 K (for x = 0.0) and 111 K (for x = 2.0, visible in the inset of Figure 8a,c), which could indicate a transition in the materials. Interestingly, the transition’s temperature for each sample remained independent of the frequency, which excludes a relaxation-type transition as the cause.

**Figure 9:**
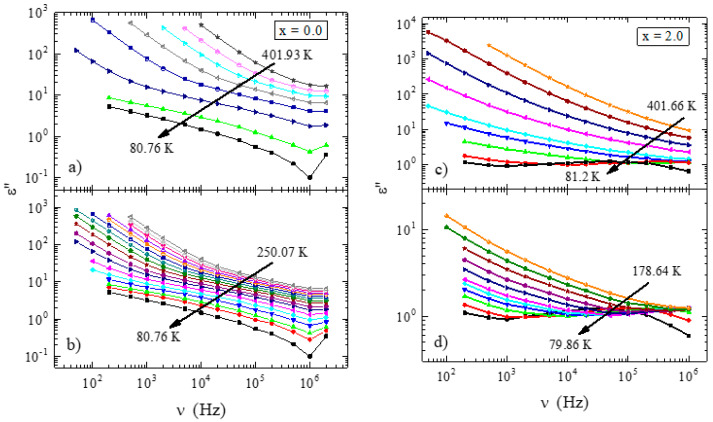
Frequency dependence of the dielectric loss spectra presented for various temperatures, ε″(v), for x = 0.0 with ΔT ≈ 50 K (20 K between two lowest temperatures) (**a**) and with ΔT ≈ 10 K (**b**), and for x = 2.0 with ΔT ≈ 50 K (10 K between two lowest temperatures) (**c**) and with ΔT ≈ 10 K (**d**). Arrows indicate the direction of the temperature evolution of the relaxation process with cooling.

**Figure 10:**
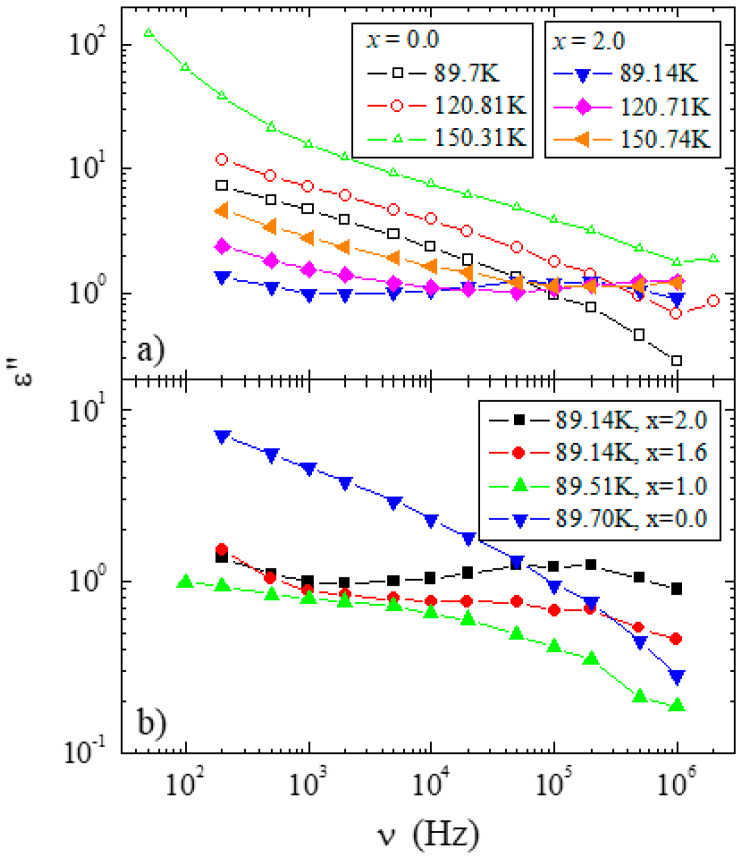
(**a**) A comparison of the dielectric loss spectra collected for the same temperatures and samples for x = 0.0 (empty symbols) and x = 2.0 (full symbols). (**b**) A comparison of the loss spectra for different samples at ≈89 K.

Because the dipole relaxation was poorly detectable with changing temperatures, the data were converted to depend on frequency. It must be mentioned that frequency dependencies of dielectric permittivity, ε′(v), and dielectric loss, ε″(v), are interrelated via Kramers–Kronig relations [37]; therefore, it is not necessary to analyze both parts of the complex dielectric permittivity, ε*(v). Consequently, only **ε**″(v) data were used for further calculations. The dielectric loss spectra for samples with x = 0.0 and 2.0 are shown in Figure 9. In this representation, two processes are visible for both samples. Linear bending with decreasing frequency was observed at higher temperatures, which shifted to lower frequencies with cooling (Figure 9a,c). This process was related to the ion conductivity of the material. Additionally, a faster relaxation process emerged with the decrease in temperature at higher frequencies, which is visible as a shoulder in the conductivity part (Figure 9b,d), and developed into a relaxation peak with further cooling (Figure 9d). This relaxation shifted to lower frequencies with cooling, which is marked using arrows in Figure 8c,d. Both processes were observed in each investigated sample; however, the degrees of development of the dipole relaxation from the conductivity part were different for different samples (see Figure 10 below).

It has to be emphasized that dipole relaxation came into the experimental frequency window at different temperatures for each sample, and for that reason, the time scale for this process should change for different samples at comparable temperatures. This was reflected in Figure 10a, where the ε″(v) spectra were compared for three similar temperatures for samples with x = 0.0 and 2.0. It is clear from this figure that the time scales of the dipole relaxation process in the material with x = 0.0 was much slower than for the sample with x = 2.0 for the same temperature conditions. As shown in Figure 10b, such a scheme was also observed for other samples: with the increasing content of dopant, the dipole relaxation became faster for the same temperature of ≈89 K. It was noticeable that the amplitude of the relaxation in the sample with x = 0.0 was about one order of magnitude higher in comparison to the others. The spectra for the sample with x = 0.4 were not compared due to the poor quality of the data for this sample in the temperature range in which the relaxation was observed. This poor quality may be related to the morphology of sample solutions, whose grain size was much larger than the grain size of the samples that were compounds.

**Figure 11:**
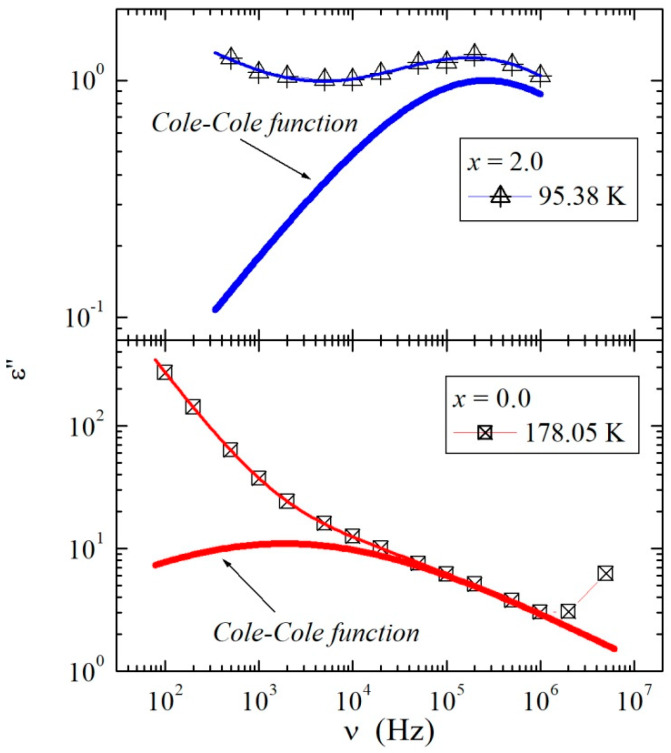
ε″(v) spectra with fitting function for samples with x = 2.0 (upper panel) and 0.0 (lower panel).

**Figure 12:**
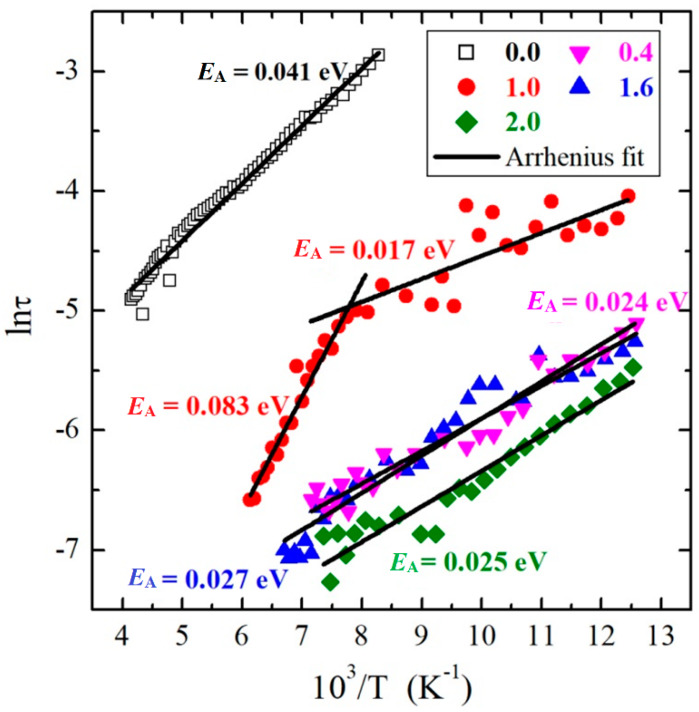
Arrhenius plot of the dielectric relaxation times, τ, for various samples. Straight (black) lines represent fits with constant activation energies, *E*_A_.

To parameterize the temperature behavior of the dipole relaxation process, the dielectric loss data, ε″(v), collected for all samples were fitted for a temperature range where the relaxation was observed using the sum of the conductivity part and the Cole–Cole equation (which describes the relaxation process visible in Figure 10) [38]:

(2) $$\varepsilon^{\prime \prime \left(\omega \right)}=Im\left(\varepsilon_{\infty }+\frac{\Delta \varepsilon }{1+{\left(i\omega \tau \right)}^{\alpha }}\right)+\frac{\sigma_{DC}}{\varepsilon_{0}\omega },$$

where σ**_DC_** is the DC conductivity, α is the shape parameter describing the symmetric broadening of the relaxation curve, Δε is the dielectric strength, and τ is the relaxation time (the analytical form of this equation used during the fitting procedure is presented in the Appendix A). Representative spectra of ε″(v) for samples with x = 0.0 and 2.0, collected at 178.05 K and 95.38 K, respectively, are presented in Figure 11, together with fitting curves described with Equation (2). The estimated relaxation times as a function of inverse temperature are presented in Figure 12. Because τ = *f* (10^3^/T), the relationships have a linear nature for samples with x = 0.0, 0.4, 1.6, and 2.0, it was possible to parameterize them using the Arrhenius equation: $\tau =\tau_{0}\exp\left(\frac{E_{A}}{kT}\right)$, where *E*_A_ is the activation energy. The estimated value of the activation energy in the intrinsic region *E*_A_ = 0.041 ± 0.001 eV for a sample with x = 0.0 was much larger in comparison with samples with x = 0.4 (*E*_A_ = 0.024 ± 0.001 eV), 1.6 (*E*_A_ = 0.027 ± 0.001 eV), and 2.0 (*E*_A_ = 0.025 ± 0.001 eV). This means that the dipole relaxation process was much more sensitive to temperature changes for this sample than for the three other materials. The above activation energy values suggest that activation of the relaxation process in the intrinsic region was much weaker than that of the electrical conductivity. Moreover, from Figure 12 and Figure 2, it is clear that the time scales of the dipole relaxation was the slowest for the sample (x = 0.0) with a higher density and was faster for Mg-doped samples with a lower density. In general, the faster the dipole relaxation, the higher the accumulation of electric charge, which requires a large energy cost. Similar behavior of dielectric relaxation in the scheelite-type Pb_1−3x_☐_x_Gd_2x_(MoO_4_)_1−3x_(WO_4_)_3x_ materials (x = 0.0455, 0.0839, 0.1154, 0.1430, 0.1667, and 0.1774, where ☐ denotes vacancies) was observed. In the above-mentioned Gd-doped lead molybdato-tungstates for poorer gadolinium samples with a lower density, the dipole relaxation was faster, while for samples richer in gadolinium with a higher density, the relaxation process was slower [39]. This finding confirms previous conclusions derived from the analysis of the data presented in Figure 10. The level of porosity of *p*_l_ ≈ 1.67%, visible in Figure 2, weakly depended on the magnesium content and had no significant effect on the relaxation processes in the materials under study.

It is important to notice that the sample with x = 1.0 shows exceptional behavior for τ(10^3^/T) (Figure 12). This may be associated with the very low residual conductivity with non-linearly increasing energy activation (Figure 3) and low electron emission at 300 and 400 K, which is visible in the I–V characteristics (Figure 5a,b). In particular, for this material, two regions with different *E*_A_’s can be distinguished. In the low-temperature range below 128 K, i.e., in an extrinsic region, *E*_A_ = 0.017 (± 0.003) eV was estimated, whereas in the high-temperature limit, i.e., in an intrinsic region, *E*_A_ = 0.084 (± 0.004) eV. This suggests that some kind of transition took place in the vicinity of T ≈ 128 K, which is not very far from the 109 K where the change in the ε_r_(T) dependence was observed for this material (see the inset in Figure 8b for x = 1.0). For another sample (excluding x = 0.0), the relaxation process was much faster. Consequently, the transitions remained invisible, most probably because it was located in the temperature range in which the relaxation process remained out of or just at the end of the experimental window. In the case of the sample with x = 0.0, the transition took place at ≈90 K (see the inset in Figure 8a for x = 0.0), and consequently, should take place for relaxation times longer than the times accessible in these experiments.

## 4. Conclusions

In summary, Zn_2−x_Mg_x_InV_3_O_11_ (*x* = 0.0, 0.4, 1.0, 1.6, and 2.0) materials were characterized in terms of electrical conductivity, thermoelectric power, I–V characteristics, and broadband dielectric spectroscopy measurements. They have shown *n*-type semiconducting properties and low electron emissions at 300 and 400 K, as well as a strong dependence on the temperature, frequency, and magnesium content of the relative dielectric constant and loss tangent. It should be highlighted that the temperature dependence of the resistivity can be very well described over a broad temperature range using the parallel resistor model. Moreover, DFT calculations showed that these materials exhibited a non-magnetic and semiconducting ground state. The width of the bandgap increased with increasing Mg content.

The dipole relaxation process in the dielectric loss spectra of Zn_2–x_Mg_x_InV_3_O_11_ was analyzed using the sum of the DC conductivity part and the Cole–Cole function. It was established that the dipole relaxation was the slowest for the sample (x = 0.0) with a higher density and became faster for Mg-doped samples (x > 0.0) with a lower density. Moreover, in the case of the sample with x = 1.0, a rather sharp change in the temperature dependence of dipole relaxation times, characterized by markedly different activation energies, was observed. It should be stressed that the faster the dipole relaxation, the greater the accumulation of electric charge. Furthermore, the relaxation time strongly depended on the magnesium content and sample density, and poorly on its porosity. The most interesting result of the studied compounds was that the weaker activation of relaxation processes made electronic transport easier in the intrinsic (semiconducting) region. The strong temperature- and frequency-dependent permittivity and high dielectric losses make these materials a poor choice as a dielectric in a typical electric capacitor. On the other hand, these materials, due to their very high thermopower over a broad temperature range and high resistivity can be used in temperature sensors that will not heat themselves due to current flow (conventional resistive sensors always heat themselves due to current flow). Thermocouples made from metallic alloys also have high thermal conductivity and much lower thermopower (at relatively low temperatures), which makes the precise measurement of the temperature of small samples difficult due to heat conduction. We see the potential application of such materials in temperature sensors used in calorimeters to measure, e.g., the electrocaloric effects at low and moderate temperatures. There exist materials with high thermopowers at low temperatures (e.g., Kondo insulators) but they do have a relatively high thermal conductivity. These compounds, due to their low symmetry and many inequivalent atomic sites, should exhibit high phonon scattering, and consequently, low thermal conductivity.

## Figures and Tables

**Table 1:** Parallel resistor model fit of Zn_2−x_Mg_x_InV_3_O_11_ (as seen in Figure 6).

x	*ρ*_0_ (Ωm)	*ρ*_p_ (10^7^ Ωm)	Δ (eV)
0	2146.7	3.95	0.154
0.4	241.5	8.46	0.444
1.0	210.4	1.07	0.517
1.6	696.7	9.97	0.306
2.0	638.8	10912	0.428

*ρ*_0_ and *ρ*_p_ are the parameters describing the two resistivities; the former changes with temperature according to the activation law, while the latter is constant with temperature. Δ is the bandgap between the valence and conduction states (see Equation (1)).

## Appendix A

The Cole–Cole function for fitting the dielectric loss relaxation process:

(3) ε″(ω)=Δε[1+2(ωτ)αsin(πα2)+(ωτ)2α]−12sinφ

where

(4) $$\varphi =arctan\left[\frac{{\left(\omega \tau \right)}^{\alpha }sin\left(\frac{\pi \alpha }{2}\right)}{1+{\left(\omega \tau \right)}^{\alpha }cos\left(\frac{\pi \alpha }{2}\right)}\right]$$
